# Extrachromosomal circular DNA promotes prostate cancer progression through the FAM84B/CDKN1B/MYC/WWP1 axis

**DOI:** 10.1186/s11658-024-00616-3

**Published:** 2024-07-12

**Authors:** Wei Jin, Zhenqun Xu, Yan Song, Fangjie Chen

**Affiliations:** 1 Department of Urology, Shengjing Hospital of China Medical University, Shenyang, 110001 Liaoning People’s Republic of China; 2 Department of Medical Genetics, China Medical University, No. 77, Puhe Road, Shenbei New District, Shenyang, 110022 Liaoning People’s Republic of China

**Keywords:** Extrachromosomal circular DNA, FAM84B, Prostate cancer, CDKN1B, MYC/WWP1

## Abstract

**Background:**

Extrachromosomal circular DNA (eccDNA), a kind of circular DNA that originates from chromosomes, carries complete gene information, particularly the oncogenic genes. This study aimed to examine the contributions of FAM84B induced by eccDNA to prostate cancer (PCa) development and the biomolecules involved.

**Methods:**

The presence of eccDNA in PCa cells and the FAM84B transcripts that eccDNA carries were verified by outward and inward PCR. The effect of inhibition of eccDNA synthesis on FAM84B expression in PCa cells was analyzed by knocking down Lig3. The impact of FAM84B on the growth and metastases of PCa cells was verified by Cell Counting Kit-8 (CCK8), EdU, transwell assays, and a xenograft mouse model. Chromatin immunoprecipitation quantitative PCR (ChIP-qPCR) and dual-luciferase reporter assays were carried out to examine the effect of FAM84B/MYC on *WWP1* transcription, and a co-immunoprecipitation (Co-IP) assay was conducted to verify the modification of CDKN1B by WWP1. The function of this molecular axis in PCa was explored by rescue assays.

**Results:**

The inhibited eccDNA synthesis significantly downregulated FAM84B in PCa cells, thereby attenuating the growth and metastasis of PCa. FAM84B promoted the transcription of WWP1 by MYC by activating the expression of MYC coterminous with the 8q24.21 gene desert in a beta catenin-dependent approach. *WWP1* transcription promoted by MYC facilitated the ubiquitination and degradation of CDKN1B protein and inversely attenuated the repressive effect of CDKN1B on MYC expression. Exogenous overexpression of CDKN1B blocked FAM84B-activated MYC/WWP1 expression, thereby inhibiting PCa progression.

**Conclusions:**

FAM84B promoted by eccDNA mediates degradation of CDKN1B via MYC/WWP1, thereby accelerating PCa progression.

**Supplementary Information:**

The online version contains supplementary material available at 10.1186/s11658-024-00616-3.

## Background

Prostate cancer (PCa) is characterized by abnormally dividing cells in the prostate gland resulting in abnormal prostate gland growth, and death from PCa mainly occurs because of metastasis when cancer cells spread to other areas of the body including the lymph nodes, the spinal cord, and the brain [1]. It has long been established that PCa is unique in its dependence on androgen for growth and progression, and androgen deprivation is an effective therapeutic strategy in clinical practice [2]. Still, identifying and utilizing biomarkers that predict survival and/or treatment response and designing optimal tools to help guide precision medicine remain the major challenges for PCa treatment [3–5].

Extrachromosomal circular DNA (eccDNA) refers to circular DNAs that originate from chromosomes, but are likely independent of chromosomal DNA once generated [6, 7]. EccDNA can carry complete gene information, particularly the oncogenic genes with increased copy number and high transcription, and the upregulation of oncogenes by eccDNA contributes to the growth of tumors [8]. For instance, more than 18,000 eccDNAs, many carrying known cancer drivers, have been identified in a pan-cancer analysis of ATAC-sequencing libraries from 23 types of tumors [9]. Regardless, the functional mechanisms of eccDNA in the progression of PCa are not clear. In the present study, we identified LRAT domain-containing 2 (*LRATD2*, also called *FAM84B*) as an eccDNA-carried gene that is upregulated in PCa using multiple bioinformatics algorithms. FAM84B has been reported to augment DU145 cell invasion and growth in soft agar and increase DU145 cell-derived xenografts and lung metastasis [10]. However, its downstream targets in PCa have not been explored. Interestingly, FAM84B has been suggested to synergize with MYC, both bordering a 1.2 Mb gene desert at 8q24.21 [11], indicating that FAM84B might interact with MYC in PCa as well. Copy number gains involving high-level amplifications at 8q21 and 8q24 have been frequently reported in breast cancer, and the breast cancer cell line SK-BR-3 contains three separate 8q21 amplicons, the distal two of which correspond to putative targets tumor protein D52 and WW domain-containing protein 1 (WWP1) [12]. In the study here, we also revealed WWP1 as one of the genes with a significant correlation with FAM84B in PCa. As a ubiquitin E3 ligase, WWP1 controlled the proteasomal destruction of many substrates [13], while its function in PCa remains unclear. This study aimed to elucidate the potential molecular mechanisms by which eccDNA-carried FAM84B promotes the progression of PCa to identify corresponding therapeutic targets.

## Materials and methods

### Patients and clinical prostate specimens

Tumor and adjacent tissues from 38 PCa patients were collected from January 2020 to December 2020. All patients were first diagnosed with PCa at Shengjing Hospital of China Medical University and did not have other malignancies. The study was conducted following the Declaration of Helsinki (as revised in 2013), and written consent for tissue donation was obtained from each patient. The protocol was approved by the Institutional Review Board of Shengjing Hospital of China Medical University (approval no. 2019PS1154K; approved date 10 June 2019).

### Cell culture, cell transfection, and establishment of stable cell lines

Human prostate epithelial cells (HPECs, cat. no. CP-H019) and PCa cell lines LNCaP (cat. no. CL-0143) and PC-3 (cat. no. CL-0185) were purchased from Procell (Wuhan, Hubei, China). All cells were cultured in RPMI-1640 medium (Gibco, Carlsbad, CA, USA) supplemented with 10% fetal bovine serum (FBS) and 1% penicillin/streptomycin at 37 °C with 5% CO_2_.

Overexpression lentivirus (oeFAM84B, oeWWP1, oeCDKN1B) based on mammalian gene expression lentiviral vector (pLV[Exp]-EGFP:T2A:Puro-EF1A), shLig3 1, 2, 3#, shWWP1 1, 2, 3# based on mammalian short hairpin (sh)RNA interference lentiviral vector (pLV[shRNA]-EGFP:T2A:Puro-U6), and their respective controls (oeCtrl and shCtrl) were purchased from VectorBuilder (Guangzhou, Guangdong, China). The virus titer was 10^9^ TU/mL, and the corresponding lentivirus was used to infect PCa cells (multiplicity of infection = 5) for 48 h. Stably transfected cell lines were subsequently screened with 2 μg/mL puromycin for 2 weeks. ShRNAs sequences specific for Lig3 and WWP1 were: shLig3 1#: CCGGATCATGTTCTCAGAAATCTCGAGATTTCTGAGAACATGATCCGG; shLig3 2#: GCCCACTTTAAGGACTACATTCTCGAGAATGTAGTCCTTAAAGTGGGC; shLig3 3#: CAGGAGTCATTAAGACTGTTTCTCGAGAAACAGTCTTAATGACTCCTG; shWWP1 1#: GACTTGAGGAGGCGCTTATATCTCGAGATATAAGCGCCTCCTCAAGTC; shWWP1 2#: CATGGAATCTGTCCGAAATTTCTCGAGAAATTTCGGACAGATTCCATG; shWWP1 3#: GCTGTTCAGAAAGGTATTAAGCTCGAGCTTAATACCTTTCTGAACAGC.

Stably transfected PCa cells were treated with MYC inhibitor MYCi361 (S8905, Selleck, Houston, TX, USA) at 6 μM for 3 h to promote MYC protein degradation [14] or Wnt signaling pathway inhibitor LF3 (S8474, Selleck) at 30 μM for 4 h to disrupt the interaction between beta catenin and TCF4. Dimethylsulfoxide (DMSO) was used as the control for both treatments.

### Outward and inward PCR

To validate the circular structure of eccDNA 3#, outward PCR primers containing different junction sites of eccDNA 3# and inward PCR primers (Table 1) targeting representative intact transcripts in different segments were designed. All reactions were performed using human genomic DNA as a control, and the PCR reaction system contained a phi29 amplification template, primers, and master mix for PCR (#1665009EDU, Bio-Rad Laboratories, Hercules, CA, USA). PCR assays were performed in a PCR cycler under standard PCR conditions according to the manufacturer’s protocols, and the circular structure of eccDNA 3# was confirmed based on agarose gel electrophoresis PCR.

**Table 1:** Primer for outward and inward PCR

Outward primer	Forward primer	Reverse primer
P1	CAAGGAGCTTTTGGGCCAAT	AGCATTGCAGTAAAAGCCAGT
P2	CGGTAAGTAGAGGAGAAAAGACG	TTGAGGCTCTGGGTGTTTCC
P3	ACAGAGCAGAGTAAATCCACCA	TCTTCCCCTGAAAATGAGCTT
**Inward primer**	**Forward primer**	**Reverse primer**
P4 (RPS26P35)	CATACAGCTTGGGAAGCTCATT	TGCACTAACTGTGCCCGAT
P5 (FAM84B)	TCTTTCCTTCAAAGAGCATCCGT	TGAAGACGGCAGTGTTGTGG

### Western blot

Total proteins were extracted from the cells using RIPA lysis buffer (Roche Diagnostics, Co., Ltd., Rotkreuz, Switzerland) containing protease and phosphatase inhibitors. Equal amounts of protein samples were separated by 8–12% sodium dodecyl sulfate–polyacrylamide gel electrophoresis and transferred to PVDF membranes (Millipore Corp, Billerica, MA, USA). Primary antibodies were incubated overnight at 4 ℃, followed by reprobing with the secondary antibody. Signals were subsequently enhanced with chemiluminescent reagents (Abcam, Cambridge, UK). Primary antibodies used were FAM84B (1:1000, 18421-1-AP, ProteinTech Group, Chicago, IL, USA), MYC (1:1000, #18583, Cell Signaling Technologies, Beverly, MA, USA), beta catenin (1:2000, 17565-1-AP, Cell Signaling Technologies), WWP1 (1:2000, 28689-1-AP, ProteinTech), CDKN1B (1:5000, ab32034, Abcam), beta actin (1:200, ab115777, Abcam), and ubiquitin (1:1000, #20326, Cell Signaling Technologies).

### PCR analysis

Total RNA was extracted from the cells using TRIzol reagent (GK20008, Glpbio, Montclair, CA, USA). After reverse transcription with the iScript cDNA Synthesis kit (#1708890, Bio-Rad Laboratories), the SsoAdvanced Universal SYBR Green Supermix (#1725270, Bio-Rad Laboratories) was used for qPCR reactions on a CFX Opus 96 Real-Time PCR system (Bio-Rad Laboratories). The relative mRNA levels were normalized using beta actin as a control. The 2^−∆∆Ct^ method was used to analyze the results. The primer sequences are presented in Table 2.

**Table 2:** Primers used for qPCR

Targets	Forward primer (5′-3′)	Reverse primer (5′-3′)
Beta actin	CACCATTGGCAATGAGCGGTTC	AGGTCTTTGCGGATGTCCACGT
Lig3	GCTACTTCAGCCGCAGTCTCAA	GCAGTGGTTTGCCTGTCTTGTTG
FAM84B	GTGGAATGCTCCGTGTTCTACC	TACTGAGCCTGCGACACGAACT
WWP1	TGAACAGTGGCAATCTCAGCGG	CTGGTGGCAAAGGTCCATAAGG
CDKN1B	ATAAGGAAGCGACCTGCAACCG	TTCTTGGGCGTCTGCTCCACAG
MYC	CCTGGTGCTCCATGAGGAGAC	CAGACTCTGACCTTTTGCCAGG
MYC promoter	AAAGAACGGAGGGAGGGATC	CTATTCGCTCCGGATCTCCC
WWP1 promoter	TTAAGAAGTCCTGCTCCGGG	TCGGATGCTGCCTTAGGAAA

WWP1: WW domain-containing protein 1; CDKN1B: cyclin-dependent kinase inhibitor 1B

### Immunohistochemistry (IHC)

Paraffin-embedded tissue sections (4 μm) were dewaxed using xylene and dehydrated with gradient ethanol, followed by the addition of 3% hydrogen peroxide to block endogenous peroxidase activity. Antigen retrieval was performed by heating sections in sodium citrate buffer (pH 6.0) in a microwave oven at 100 °C for 30 min, followed by detection of immunoreactivity by the Rabbit-diaminobenzidine Detection IHC kit (IHC0007, FineTest, Wuhan, Hubei, China). Briefly, nonspecific antigen binding was blocked by blocking serum, and then the sections were stained with primary antibody overnight at 4 °C, followed by color development by DAB after incubation with poly-horseradish peroxidase-Goat Anti-Rabbit IgG at room temperature for 1 h. Hematoxylin was used to counter-stain the nuclei. Scoring was performed for clinical samples and was completed by three pathologists who were unaware of the grouping. The score was determined according to the positive staining intensity (0: negative; 1: weak; 2: moderate; 3: strong) and positive staining cells quantity: (0: < 5%; 1: 5~25%; 2: 25~50%; 3: 50~75%; 4: > 75%), and the final score was intensity score × quantity score (0–12). Xenograft tumor tissues were viewed by microscopy, and positively stained areas were quantified using Image J software. Antibodies to FAM84B (1:200, 18421-1-AP, ProteinTech), Ki67 (1:200, 28074-1-AP, ProteinTech), WWP1 (1:500, 28689-1-AP, ProteinTech), Lig3 (1:200, A22136, ABclonal, Wuhan, Hubei, China), MYC (1:200, #18583, Cell Signaling Technologies), and CDKN1B (1:50, ab32034, Abcam) were used.

### Proliferation and viability assays

The treated PCa cells were suspended with 100 μL of the medium, seeded into 96-well plates (3000 cells/well), and incubated for the indicated periods (1, 3, 5 days). After the addition of 10 μL of Cell Counting Kit-8 (CCK8; GK10001, Glpbio) to each well, the incubation continued for 2 h in a cell incubator. The cell proliferation was measured by reading the OD value at 450 nm using a microplate reader.

The DNA synthesis of the cells was determined using the BeyoClick EdU-594 Cell Proliferation Assay kit (C0078S, Beyotime, Shanghai, China) according to the supplier’s protocol. Treated PCa cells (1 × 10^4^) were placed in a 96-well plate and treated with 50 μM of EdU for 2 h. Afterward, the cells were fixed in 4% paraformaldehyde in PBS for 30 min at room temperature and permeabilized with 0.5% TritonX-100 for 10 min, followed by staining with Click Additive Solution for 30 min in the dark. Hoechst 33342 was used to stain cells for 5 min in the dark to label the nucleus. Finally, the cells were observed by fluorescence microscopy (Olympus, Tokyo, Japan), and the proportion of EdU-positive cells was calculated.

### Migration and invasion assays

Transwell chambers (8-μm pore size, Corning Costar, Corning, NY, USA) were used for cell migration and invasion assays. For the migration assay, 1 × 10^5^ treated PCa cells were seeded in the apical chamber containing serum-free medium, and a complete medium containing 10% FBS was supplemented to the basolateral chamber. After 24 h, migrated cells were fixed with 4% paraformaldehyde and stained with crystal violet. For the invasion assay, the apical chamber was precoated with Matrigel, and the rest of the steps were the same as for the migration assay.

### Chromatin immunoprecipitation (ChIP) assay

The promoter sequence of MYC (chr8:127736036–127736381) and the WWP1 promoter sequence (chr8:86341669–86343512) with a potential binding relationship to MYC were obtained from the UCSC Genome Browser (https://genome.ucsc.edu/index.html). The SimpleChIP Plus Enzymatic ChIP kit (#9005, Cell Signaling Technologies) was used to detect the ability of MYC to recruit the WWP1 promoter and and the ability of beta catenin to recruit the MYC promoter. The cells were fixed with formaldehyde and then lysed, and chromatin was partially digested with micrococcal nuclease to form fragments. The chromatin fractions were incubated with antibodies to one of the following: MYC (1:100, #18583, Cell Signaling Technologies), beta catenin (1:100, 17565-1-AP, Cell Signaling Technologies), or normal rabbit IgG with ChIP-grade protein G magnetic beads. After protein–DNA was de-crosslinked, purification was performed using DNA purification centrifuge columns, and enrichment of the WWP1 promoter or MYC promoter was detected by qPCR reaction. The qPCR reaction system consisted of nuclease-free H_2_O, 5 µM promoter primer, and SimpleChIP universal qPCR premix. A total of 40 cycles of the standard PCR reaction program were performed according to the manufacturer’s protocol. The signal obtained from each immunoprecipitation was expressed as a percentage of the total input chromatin: % of input = 1% × 2^(Ct 1%input sample−Ct IP sample)^, Ct = Threshold cycle of PCR reaction.

### Luciferase reporter assay

The WWP1 promoter sequence (chr8:86341669–86343512), which has a binding relationship with MYC or the MYC promoter sequence (chr8:127736036–127736381) was inserted into the pGL3 basic vector (Promega Corporation, Madison, WI, USA) to construct the WWP1 or MYC promoter luciferase reporter plasmid. These plasmids were transfected into treated PCa cells by Lipofectamine 2000 (Thermo Fisher Scientific Inc., Waltham, MA, USA), and the luciferase activity was assessed by a dual luciferase reporter assay system (Promega) after 48 h.

### TOP/FOP flash

TOP flash plasmid (D2501, Beyotime) containing the TCF/LEF binding site was used to detect Wnt/β-catenin pathway activity in cells, and the FOP flash plasmid (D2503, Beyotime) containing the mutated TCF/LEF binding site sequence was used as a negative control. The above plasmids were transfected into the treated PCa cells by Lipofectamine 2000 (Thermo Fisher), and the luciferase activity was measured by Dual-Luciferase Reporter Analysis System (Promega Corporation, Madison, WI, USA) after 48 h.

### Formation of xenografts and metastases

The protocols were approved by the Animal Research Ethics Board of Shengjing Hospital of China Medical University (approval no. 2022PS1170K; approved date 4 January 2022) following the Guidelines for the Care and Use of Animals. The study involving animals was conducted following the Basel Declaration. Seven-week-old male NOD/SCID mice were purchased from Beijing Vital River Laboratory Animal Technology Co., Ltd. (Beijing, China) and acclimatized for 1 week before the experiment. All mice were randomly divided into 12 groups of 10 mice each.

Five mice in each group were randomly selected for tumor growth experiments. The PC-3 cells were suspended in PBS at a density of 1 × 10^7^ cells/mL, and Matrigel (Corning) was added to the cell suspension at a ratio of 1:1. The mixture (150 μL) was injected subcutaneously into the back of mice to induce tumor growth. The tumor volume (V) was measured once a week with calipers, and the formula is as follows: *V* = 0.5 × L × W^2^, where L = length and W = width. After 4 weeks, the mice were euthanized, and their tumor size and weight were measured.

The remaining five mice in each group were used for tumor metastasis experiments. PC-3 cells were labeled with luciferase and treated as indicated. Subsequently, 100 µL (1 × 10^7^ cells/mL) of the cell suspension was injected intracardially into the left ventricle. Each week, 150 µL of 30 mg/mL d-luciferin was injected intraperitoneally into each mouse, and then luciferase activity was measured using the IVIS Small Animal Live Imaging System to assess tumor metastasis. For mice requiring MYCi361 treatment, MYCi361 (55 mg/kg/day) was administered by gavage after tumor cell injection for 3 consecutive days per week for 2 weeks.

### Co-immunoprecipitation (Co-IP)

PCa cells in the oeCtrl and oeWWP1 groups were pretreated with MG-132 (S2619, Selleck) at a concentration of 100 nM for 12 h to inhibit 26S proteasome-mediated protein degradation, followed by lysis in RIPA lysis buffer and centrifugation. A portion of the supernatant was used as input, and the other supernatant was immunoprecipitated with CDKN1B antibody (1:30, ab32034, Abcam) overnight at 4 °C and incubated with protein A/G agarose beads (Thermo Fisher Scientific) for 60 min at room temperature. The immune complexes were washed with RIPA buffer containing 5% Tween-80 and assayed by western blot assays.

### Determination of protein stability

Cycloheximide (CHX, S7418, Selleck) was used to treat PCa cells in the oeCtrl and oeWWP1 groups at a concentration of 500 nM for 0, 3, and 6 h to inhibit protein synthesis. The cells were collected at each time point, and CDKN1B protein expression was detected by western blot assays to assess the protein stability of CDKN1B.

### Data analysis

Experiments were carried out at least three times unless otherwise indicated. Statistical analysis was performed using GraphPad Prism 8.0.2 software (GraphPad, San Diego, CA, USA). All data were described as mean ± standard deviation (SD) if applicable. Statistical difference was performed with paired *t*-test (two groups) or one-way/two-way analysis of variance (ANOVA) (three or more groups), followed by Tukey’s or Dunnett’s post hoc test, where appropriate. For the correlation between genes, data were compared using Pearson’s correlation analysis, and for the correlation between gene expression in PCa tissues and clinical parameters in patients, a Chi-square test was conducted. *p*-value < 0.05 was considered statistically significant.

## Results

### FAM84B carried by eccDNA is upregulated in PCa

eccDNAdb: a database of eccDNA profiles in human cancers (http://www.eccdnadb.org/searchbyeccdna/?) analyzed whole-genome sequencing data derived from a total number of 3395 tumor samples, and 1270 eccDNAs from 480 samples were detected and included in the database. EccDNA present in PCa samples was filtered to exclude eccDNA with unavailable data (a copy count of −1) (Supplementary Table 1), and three sequences of nonrepetitive eccDNA were detected in PCa (Fig. S1A). Meanwhile, the eccDNAdb database showed that eccDNA 1#, 2# did not contain complete gene transcripts, while Segment 1 and Segment 2 of eccDNA 3# contained multiple complete gene transcripts (Supplementary Table 2). The expression of eccDNA-carried genes with complete transcripts contained in eccDNA 3# in PCa was analyzed using the eccDNAdb database. LRATD2 (FAM84B) had the highest abundance (fragments per kilobase of exon per million mapped reads) in PCa, and its expression was most significantly elevated in tumor tissues compared to normal tissues (Fig S1B). GEPIA (http://gepia.cancer-pku.cn/index.html) is a newly developed interactive web server for analyzing the RNA sequencing expression data of 9736 tumors and 8587 normal samples from the TCGA and the GTEx projects, using a standard processing pipeline. Further validating the expression and the prognostic significance of FAM84B in PCa in GEPIA, we observed that FAM84B expression was significantly elevated in PCa and predicted a poor prognosis for patients (Fig. S1C, D). Therefore, eccDNA3# was selected for validation in PCa cells.

**Fig. 1:**
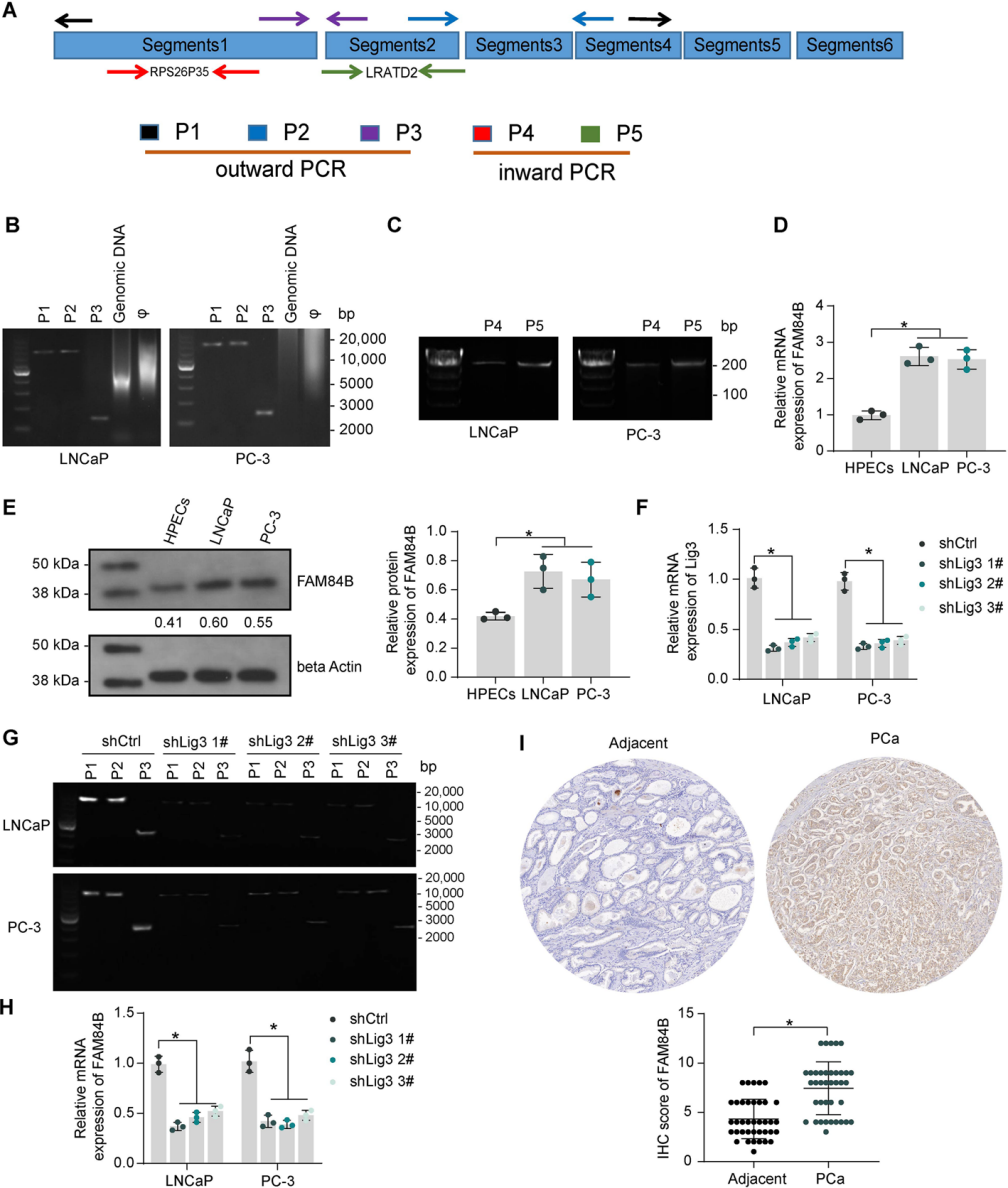
Overexpression of FAM84B in PCa is induced by eccDNA. **A** PCR primer design for the validation of eccDNA circular structures and embedded transcripts. **B** Outward PCR validation of eccDNA circular structure. **C** Inward PCR detection of eccDNA-containing transcripts. **D**–**E** RT–qPCR and western blot detection of FAM84B expression in PCa cells and HPECs. **F** The efficiency of shRNAs targeting Lig3 was measured using RT–qPCR. **G** Effect of shLig3 on eccDNA 3# levels in PCa cells was measured using outward PCR. **H** Effect of knockdown of Lig3 on FAM84B expression in cells by RT–qPCR. **I** FAM84B protein expression in PCa and adjacent tissues (*n* = 38) was examined using IHC. Experiments were repeated three times with multiple wells. The bars indicate SD. *p < 0.05 (paired *t*-test, one-way/two-way ANOVA)

Outward PCR primers containing different junction sites of eccDNA 3# and inward PCR primers for representative complete transcripts in different segments were designed (Fig. 1A). We confirmed the presence of circular DNA structures by nucleic acid exonuclease phi29 (φ) treatment and outward PCR in the androgen-sensitive PCa cell line LNCaP (AR-positive) as well as in the androgen-resistant PCa cell line PC-3 (AR-negative) (Fig. 1B). Inward PCR (Fig. 1C) showed that the RPS26935 and LRATD2 transcripts were contained in eccDNA 3#.

Compared to HPECs, the mRNA and protein expression of FAM84B was significantly higher in both LNCaP and PC-3 cells (Fig. 1D, E), suggesting that FAM84B expression is not associated with AR status or androgen sensitivity. DNA ligases are crucial for most DNA transactions, including DNA replication, repair, and recombination [15]. DNA ligase III (Lig3) has been demonstrated to be crucial to eccDNA generation [16]. We infected PCa cell lines with lentivirus of shRNA targeting Lig3 and confirmed the knockdown efficiency (Fig. 1F) by RT–qPCR. Cells were analyzed for eccDNA 3# by outward PCR containing eccDNA 3# at different junction sites. We found that inhibition of DNA fragment cyclization by shLig3 leads to almost undetectable valid bands by outward PCR in cells, with a significant reduction in eccDNA 3# levels (Fig. 1G). Meanwhile, the expression of FAM84B was significantly reduced, suggesting that eccDNA mediates the amplification of FAM84B gene expression (Fig. 1H). A significant increase in the expression of FAM84B was observed in collected PCa tissues relative to adjacent tissues, as revealed by IHC (Fig. 1I). PCa patients were divided into two cohorts of high FAM84B expression (*n* = 23) and low FAM84B expression (*n* = 15) based on the mean FAM84B IHC scores in tumor tissues. High expression of FAM84B in tumor tissues was significantly correlated with higher Gleason score and T stage in patients, but not with patients’ age, serum PSA content, molecular subtype (AR, p53), and lymph node metastasis (Table 3).

**Table 3:** Correlation between FAM84B expression in tumor tissues and clinical characteristics of PCa patients

Clinical characteristics		*n* = 38	FAM84B IHC score		*p*-Value
			High (*n* = 23)	Low (*n* = 15)	
Age (years)	≥ 66	17	12	5	0.2536
	< 66	21	11	10	
PSA (ng/mL)	≥ 9.67	13	7	6	0.5435
	< 9.67	25	16	9	
Gleason score	≤ 6	11	1	10	0.0003*
	3 + 4	10	7	3	
	4 + 3	9	7	2	
	≥ 8	8	8	0	
AR statue	Positive	30	18	12	0.8977
	Negative	8	5	3	
p53 statue	Positive	15	10	5	0.5317
	Negative	23	13	10	
T stage	pT2	23	9	14	0.0037*
	pT3	13	12	1	
	pT4	2	2	0	
Lymph node metastasis	Absent	27	15	12	0.326
	Present	11	8	3	

Chi-square test was used to analyze the correlation between gene expression in PCa tumor tissues and clinical characteristics of patients. PCa: prostate cancer; PSA: prostate-specific antigen; AR: androgen receptor; IHC: immunohistochemistry; *p < 0.05

### eccDNA-mediated overexpression of FAM84B promotes malignant progression of PCa cells

**Fig. 2:**
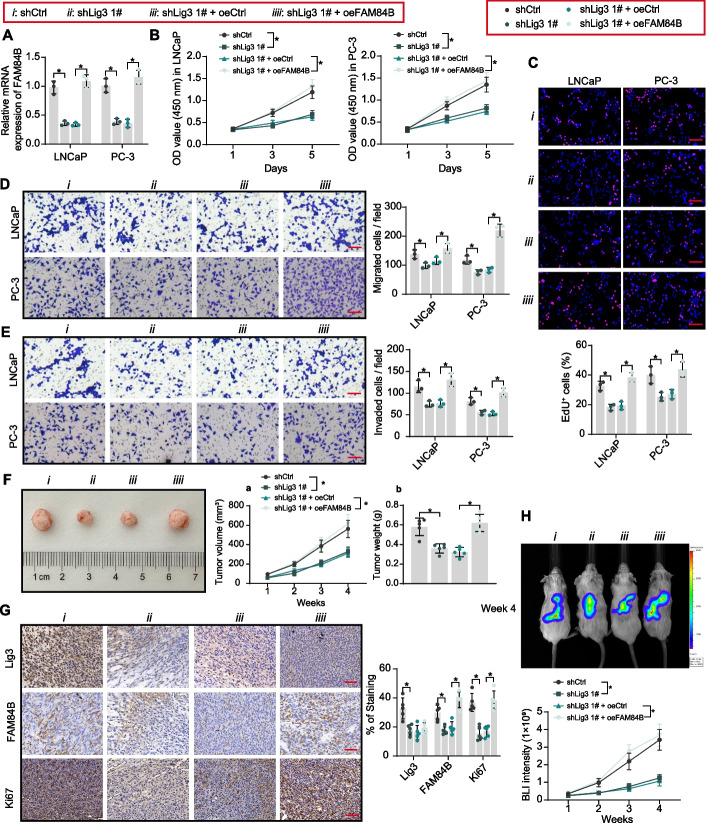
FAM84B overexpression promotes the growth and metastasis of PCa cells. PCa cells were infected with shCtrl, shLig 3 1#, shLig 3 1# + oeCtrl, shLig 3 1# + oeFAM84B. **A** Detection of FAM84B expression by RT–qPCR. **B**, **C** The PCa cell proliferation and DNA synthesis activity were assessed using CCK8 and EdU staining. **D**, **E** The migratory and invasion of PCa cells were assessed using transwell assays. **F** The growth rate of xenograft tumors formed by subcutaneously inoculated PC-3 cells in mice (*n* = 5). **G** Lig3, FAM84B, and Ki67 protein expression in xenograft tumors (*n* = 5) was examined using IHC. **H** Observation of tumor cell metastasis formed by intracardiac injection of PC-3 cells by bioluminescence imaging (*n* = 5). Experiments were repeated three times with multiple wells. The bars indicate SD. *p < 0.05 (one-way/two-way ANOVA). Scale bar = 50 μm

FAM84B was overexpressed by lentiviral infection in PCa cell lines with knockdown of Lig3, and lentiviral overexpression of FAM84B significantly reversed the downregulation of FAM84B induced by knockdown of Lig3 (Fig. 2A). Knockdown of Lig3 significantly inhibited PCa cell proliferation and DNA synthesis, whereas overexpression of FAM84B significantly rescued PCa cell activity (Fig. 2B, C). It was observed using the transwell assay that knockdown of Lig3 inhibited cell migration and invasion, while overexpression of FAM84B activated cell metastatic activity (Fig. 2D, E).

More malignant PC-3 cells were used to perform xenograft experiments. Subcutaneous injection of PC-3 cells was used to induce tumor growth, and knockdown of Lig3 inhibited the growth of xenograft tumors and reduced tumor weight, which was reversed by overexpression of FAM84B (Fig. 2F). IHC confirmed reduced FAM84B expression by Lig3 inhibition in xenograft tumors and reduced expression of the proliferation marker Ki67. However, FAM84 overexpression induced Ki67 expression (Fig. 2G). PC-3 cells were intracardially injected into mice to construct an in vivo metastasis model, and the knockdown of Lig3 attenuated tumor load and reduced distant metastasis. By contrast, overexpression of FAM84B showed metastasis-promoting properties (Fig. 2H).

### FAM84B promotes the expression of WWP1 in PCa

**Fig. 3:**
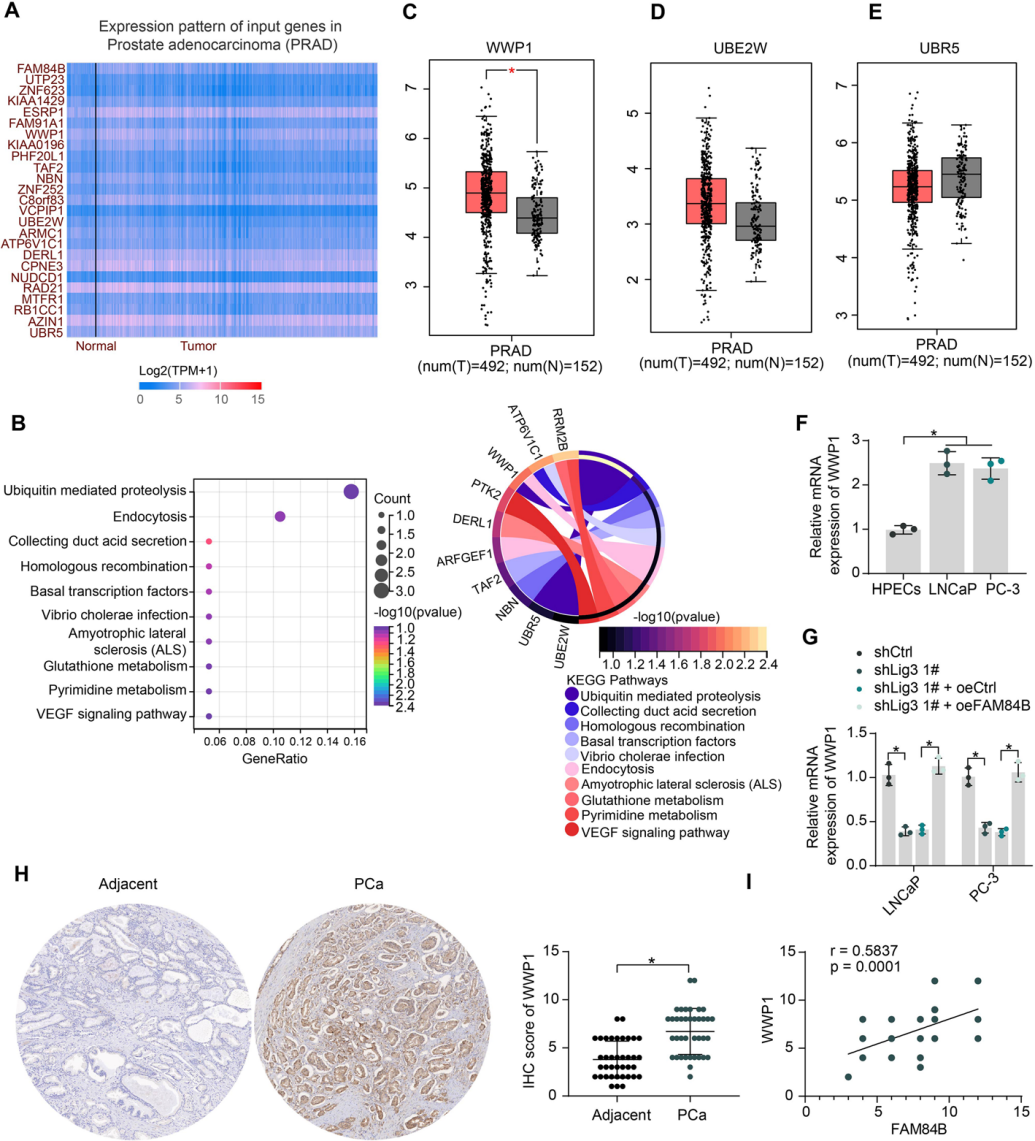
Downstream target analysis of FAM84B. **A** The heatmap of the top 25 genes significantly and positively correlated with FAM84B expression. **B** Pathway enrichment analysis of FAM84B co-expressed genes. GEPIA analysis of expression of WWP1 (**C**), UBE2W (**D**), and UBR5 (**E**) in PCa. **F** The expression of WWP1 in PCa cells and HPECs was assessed using RT–qPCR. **G** Effect of knockdown of Lig3 and overexpression of FAM84B on WWP1 mRNA expression by RT–qPCR. **H** WWP1 protein expression in PCa and adjacent tissues (*n* = 38) was examined using IHC. **I** The correlation of WWP1 expression and FAM84B expression in PCa tissues was analyzed using Pearson’s correlation analysis (*n* = 38). Experiments were repeated three times with multiple wells. The bars indicate SD. *p < 0.05 (paired *t*-test, one-way/two-way ANOVA)

UALCAN (https://ualcan.path.uab.edu/cgi-bin/ualcan-res.pl) is a comprehensive, user-friendly, and interactive web resource for analyzing cancer OMICS data. To analyze transcriptome changes in PCa affected by eccDNA-mediated FAM84B expression, we downloaded genes co-expressed with FAM84B in PCa from UALCAN. The heatmap in Fig. 3A shows the top 25 genes in terms of the correlation coefficient. The genes with correlation coefficients greater than or equal to 0.7 (Supplementary Table 3) were subjected to Kyoto Encyclopedia of Genes and Genomes (KEGG) pathway enrichment analysis, and we observed that genes co-expressed with FAM84B were significantly involved in the hsa04120: Ubiquitin mediated proteolysis pathway and three genes co-expressed with FAM84B were present in this pathway: WWP1, UBE2W, and UBR5 (Fig. 3B). Analysis in GEPIA showed that the expression of WWP1 (Fig. 3C) was significantly increased in PCa, while the expression of UBE2W and UBR5 was not significantly altered in PCa (Fig. 3D, E).

RT–qPCR experiments showed that WWP1 expression was significantly elevated in the PCa cell lines (Fig. 3F). Lig3 knockdown inhibited WWP1 expression in the PCa cell lines, while WWP1 expression was significantly restored after overexpression of FAM84B (Fig. 3G). In addition, we also detected high expression of WWP1 in PCa tissues (Fig. 3H), and its expression was significantly and positively correlated with FAM84B (Fig. 3I). PCa patients were divided into two cohorts of high WWP1 expression (*n* = 19) and low WWP1 expression (*n* = 19) based on the mean WWP1 IHC scores in the tumor tissues (Table 4). The correlation between WWP1 expression in tumor tissues and clinical parameters of patients was analyzed, and it was observed that high expression of WWP1 was associated with higher Gleason score and T stage in patients.

**Table 4:** Correlation between WWP1 expression in tumor tissues and clinical characteristics of PCa patients

Clinical characteristics		*n* = 38	WWP1 IHC score		*p*-Value
			High (*n* = 19)	Low (*n* = 19)	
Age (years)	≥ 66	17	11	6	0.1028
	< 66	21	8	13	
PSA (ng/mL)	≥ 9.67	13	7	6	0.7324
	< 9.67	25	12	13	
Gleason score	≤ 6	11	2	9	0.0037*
	3 + 4	10	3	7	
	4 + 3	9	8	1	
	≥ 8	8	6	2	
AR status	Positive	30	15	15	> 0.9999
	Negative	8	4	4	
p53 status	Positive	15	8	7	0.7400
	Negative	23	11	12	
T stage	pT2	23	7	16	0.0076*
	pT3	13	11	2	
	pT4	2	1	1	
Lymph node metastasis	Absent	27	14	13	0.7206
	Present	11	5	6	

Chi-square test was used to analyze the correlation between gene expression in PCa tumor tissues and clinical characteristics of patients. PCa: prostate cancer; PSA: prostate-specific antigen; AR: androgen receptor; IHC: immunohistochemistry; *p < 0.05

### MYC is involved in the regulatory role of FAM84B on WWP1

To investigate the molecular mechanism of FAM84B-mediated WWP1, we first analyzed the function of FAM84B. The UCSC Genome Browser (https://genome.ucsc.edu/) is a web-based tool serving as a multipowered microscope that allows researchers to view all 23 chromosomes of the human genome at any scale from a full chromosome down to an individual nucleotide. As predicted by the UCSC Genome Browser, FAM84B was localized to the 8q24.21 gene desert (Fig. S2A). FAM84B has been suggested to contribute to the oncogenic effect of MYC, and the FAM84B and MYC genes border a 1.2 Mb gene desert at 8q24.21 [11]. By GEPIA, we also verified the positive correlation between MYC and FAM84B or WWP1 in PCa (Fig S2B, C). The Cistrome Data Browser (http://cistrome.org/db/#/) is a resource of ChIP-seq, ATAC-seq, and DNase-seq data from humans and mice and provides maps of the genome-wide locations of transcription factors, cofactors, chromatin remodelers, histone posttranslational modifications, and regions of chromatin accessible to endonuclease activity. We observed a significant binding of MYC to the WWP1 promoter in LNCaP cells from the ChIP-seq database in the Cistrome Data Browser (Fig. S2D). We therefore hypothesized that FAM84B-mediated MYC activates WWP1 transcription.

**Fig. 4:**
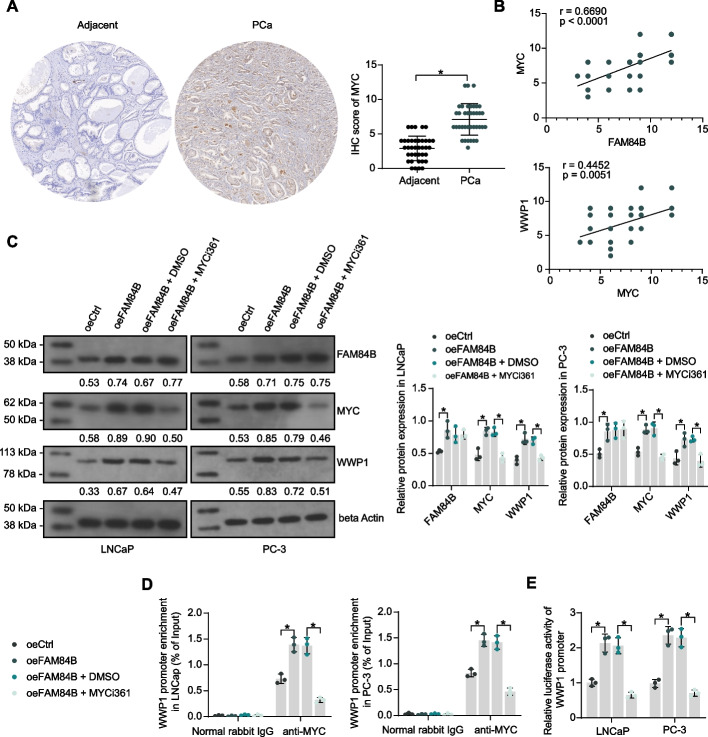
FAM84B-mediated MYC activates the transcription of WWP1. **A** MYC protein expression in PCa and adjacent tissues (*n* = 38) was examined using IHC. **B** The correlation of MYC expression and FAM84B/WWP1 expression in PCa tissues was analyzed using Pearson’s correlation analysis (*n* = 38). **C** The FAM84B, MYC, and WWP1 protein expression in PCa cells in response to overexpression of FAM84B and MYC inhibitor MYCi361 treatment was examined using western blot assays. **D** The binding ability of MYC in the WWP1 promoter region in PCa cells in response to overexpression of FAM84B and MYC inhibitor MYCi361 treatment was examined using western blot assays. **E** The transcription of WWP1 promoter in PCa cells in response to overexpression of FAM84B and MYC inhibitor MYCi361 treatment was analyzed using dual-luciferase assays. Experiments were repeated three times with multiple wells. The bars indicate SD. *p < 0.05 (paired *t*-test, two-way ANOVA)

MYC was highly expressed in PCa tissues (Fig. 4A) and was significantly and positively correlated with FAM84B and WWP1 expression (Fig. 4B). PCa patients were divided into two cohorts of high MYC expression (*n* = 19) and low MYC expression (*n* = 19) based on the mean MYC IHC scores in tumor tissues. Higher expression of MYC in tumor tissues was associated with higher Gleason score, p53 status, and lymph node metastasis in patients (Table 5). The prognostic significance of MYC on patients’ clinical characteristics differed from that of FAM84B and WWP1, which we hypothesized to be related to the small number of our clinical samples (*n* = 38).

**Table 5:** Correlation of MYC expression in tumor tissues with clinical parameters of PCa patients

Clinical characteristics		*n* = 38	MYC IHC score		*p*-Value
			High (*n* = 19)	Low (*n* = 19)	
Age (years)	≥ 66	17	8	9	0.7442
	< 66	21	11	10	
PSA (ng/mL)	≥ 9.67	13	6	7	0.7324
	< 9.67	25	13	12	
Gleason score	≤ 6	11	1	10	0.0012*
	3 + 4	10	6	4	
	4 + 3	9	4	5	
	≥ 8	8	8	0	
AR status	Positive	30	13	17	0.1115
	Negative	8	6	2	
p53 status	Positive	15	11	4	0.0202*
	Negative	23	8	15	
T stage	pT2	23	10	13	0.2911
	pT3	13	7	6	
	pT4	2	2	0	
Lymph node metastasis	Absent	27	10	17	0.0123*
	Present	11	9	2	

Chi-square test was used to analyze the correlation between gene expression in PCa tumor tissues and clinical characteristics of patients. PCa: prostate cancer; PSA: prostate-specific antigen; AR: androgen receptor; IHC: immunohistochemistry; *p < 0.05

PCa cell lines stably overexpressing FAM84B were constructed and treated with the MYC inhibitor MYCi361 in combination. It was found using Western blot that FAM84B overexpression significantly increased the expression of MYC and WWP1, while MYCi361 significantly reversed the effect of FAM84B (Fig. 4C). Chromatin immunoprecipitation quantitative PCR (ChIP-qPCR) experiments showed that overexpression of FAM84B resulted in increased MYC enrichment on the WWP1 promoter, while MYC enrichment to the WWP1 promoter fragment was significantly reduced after MYCi361 treatment (Fig. 4D). Finally, overexpression of FAM84B promoted the transcription of the WWP1 promoter, as demonstrated by promoter luciferase reporter assays, while simultaneous treatment with MYCi361 significantly inhibited the transcription of the WWP1 promoter (Fig. 4E).

The exact mechanism by which FAM84B mediates MYC activation remains unclear. Distal enhancer on 8q24 mediates MYC transcription through beta catenin/TCF4 signaling [17], and silencing of FAM84B markedly reduced the level of active beta catenin and the transcription activity of TCF/LEF in glioma [18]. We therefore hypothesized that MYC expression in FAM84B-activated PCa might be beta catenin-dependent. PCa cells overexpressing FAM84B were treated with the classical Wnt signaling pathway inhibitor LF3 to disrupt the interaction between beta catenin and TCF4. Western blot detected that overexpression of FAM84B enhanced the protein expression of beta catenin in PCa cells, whereas LF3 did not affect the protein expression of beta catenin (Fig. S3A). The TOP/FOP flash assay confirmed that overexpression of FAM84B enhanced beta catenin signaling in PCa cells and LF3 significantly blocked beta catenin signaling (Fig. S3B). RT–qPCR showed that overexpression of FAM84B promoted the transcriptional expression of MYC, and blocking beta catenin signaling using LF3 reduced MYC transcription (Fig. S3C). ChIP–qPCR experiments detected that overexpression of FAM84B promoted the enrichment of beta catenin at the MYC promoter, while LF3 treatment inhibited beta catenin binding to the MYC promoter (Fig. S3D). The results of the MYC promoter luciferase reporter assay demonstrated that FAM84B promoted MYC promoter transcriptional activity in a beta catenin-dependent manner (Fig. S3E).

### MYC shows the tumor-promoting and metastasis-promoting properties in PCa in a WWP1-dependent manner

**Fig. 5:**
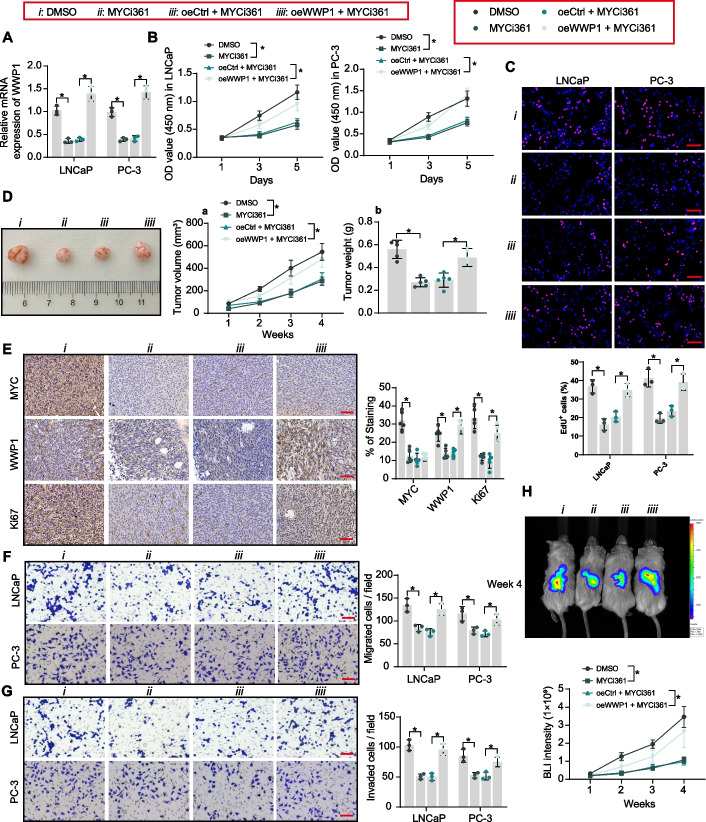
MYC promotes WWP1 expression to drive PCa tumor progression. PCa cells were treated with DMSO, MYCi361, oeCtrl + MYCi361, and oeWWP1 + MYCi361 (**A**) WWP1 mRNA expression in PCa cells was examined using RT–qPCR. **B**, **C** The PCa cell proliferation and DNA synthesis activity were assessed using CCK8 and EdU staining. **D** The growth rate of xenograft tumors formed by subcutaneously inoculated PC-3 cells in mice treated with MYCi361 or DMSO (*n* = 5). **E** MYC, WWP1, and Ki67 protein expression in xenograft tumors (*n* = 5) was examined using IHC. **F**, **G** The migratory and invasive capacity of PCa cells were examined using transwell assays. **H** Observation of tumor cell metastasis formed by intracardiac injection of PC-3 cells in mice treated with MYCi361 or DMSO by bioluminescence imaging (*n* = 5). Experiments were repeated three times with multiple wells. The bars indicate SD. *p < 0.05 (one-way/two-way ANOVA). Scale bar = 50 μm

MYC inhibitor MYCi361 was used to treat PCa cell lines with or without oeWWP1. RT–qPCR detected that MYCi361 repressed the transcription of endogenous WWP1, but oeWWP1-mediated exogenous WWP1 overexpression significantly reversed the effect of MYCi361 on WWP1 expression (Fig. 5A). This again validates that MYC activates WWP1 transcription by binding to the WWP1 promoter, as MYCi361 is unable to block WWP1 transcription mediated by an exogenous overexpressing lentivirus using the strong promoter (EF1A).

MYCi361 treatment significantly inhibited the proliferation of PCa cells and suppressed DNA synthesis, while the overexpression of WWP1 rescued PCa cell growth in vitro (Fig. 5B, C). In in vivo experiments, tumor growth inhibition by MYCi361 administration was also observed, but the inhibitory effect of MYCi361 on the growth of tumor cells was significantly attenuated following WWP1 overexpression (Fig. 5D). IHC detected that MYCi361-mediated MYC inhibition reduced the expression of WWP1 and Ki67 in xenograft tumors. However, Ki67 expression was enhanced in tumor tissues stably overexpressing WWP1 (Fig. 5E).

MYCi361 treatment inhibited the migratory and invasive properties of PCa cells (Fig. 5F, G) and reduced the metastatic dissemination in vivo (Fig. 5H). In contrast, the inhibitory effect of MYCi361 on the metastatic activity of PCa cells was significantly reversed by oeWWP1 (Fig. 5F–H).

### WWP1 mediates the degradation of CDKN1B

**Fig. 6:**
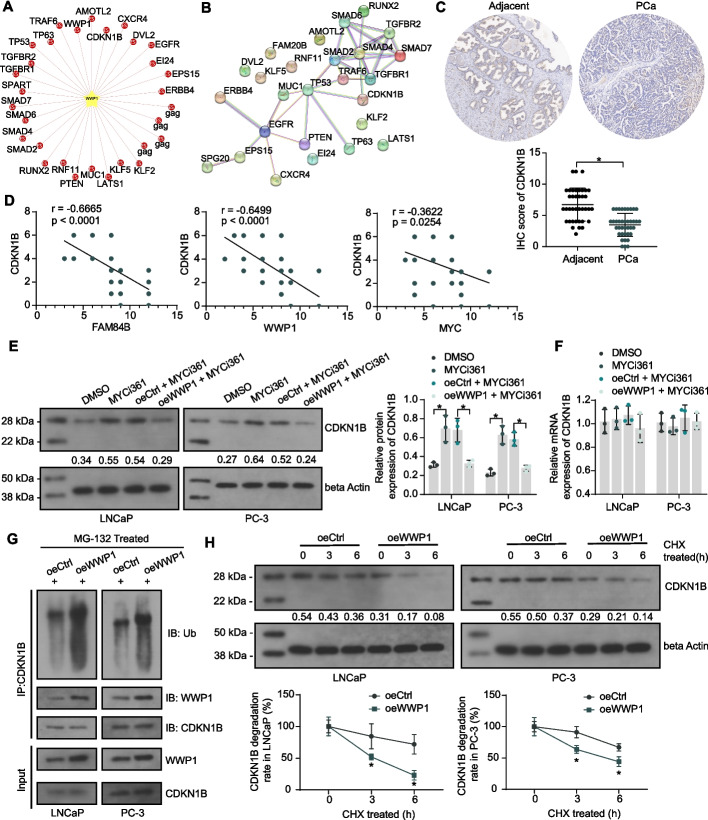
Downstream target analysis of WWP1. **A** Predicted downstream target proteins of E3 ubiquitin ligase WWP1. **B** PPI network of WWP1 target proteins. **C** CDKN1B protein expression in PCa tissues and adjacent tissues (*n* = 38) was examined using IHC. **D** The correlation of CDKN1B expression and FAM84B/WWP1/MYC expression in PCa tissues was analyzed using Pearson’s correlation analysis (*n* = 38). **E** The CDKN1B protein expression in PCa cells in response to overexpression of WWP1 and MYC inhibitor MYCi361 treatment was examined using western blot assays. **F** The CDKN1B mRNA expression in PCa cells in response to overexpression of WWP1 and MYC inhibitor MYCi361 treatment was examined using RT–qPCR. **G** Endogenous interaction between CDKN1B and WWP1 in PCa cells and the effect of overexpression of WWP1 on ubiquitination of CDKN1B protein examined using Co-IP. **H** The effect of overexpression of WWP1 on the stability of CDKN1B protein in PCa cells treated with CHX. Experiments were repeated three times with multiple wells. The bars indicate SD. *p < 0.05 (one-way/two-way ANOVA)

UbiBrowser 2.0 (http://ubibrowser.bio-it.cn/ubibrowser_v3/) is a comprehensive resource for proteome-wide known and predicted ubiquitin ligase (E3)/deubiquitinase–substrate interactions in eukaryotic species. For the analysis of the WWP1-mediated cancer promotion mechanism, we first predicted the downstream proteins of WWP1 (Fig. 6A) in UbiBrowser 2.0. The STRING database (https://string-db.org/) systematically collects and integrates protein–protein interactions (PPI) under physical and functional associations. The PPI network of WWP1 targets was constructed using STRING (Fig. 6B). In the network, there are known tumor suppressors in PCa, such as CDKN1B [19], TP53 [20], and PTEN [21]. Considering that WWP1 exhibited oncogenic effects in PC-3 cells [22] that do not express TP53 (p53 null), TP53 is not a major target of action of WWP1. Meanwhile, CDKN1B has been reported to inhibit MYC expression in PCa [23]. This suggests that MYC mediates the transcription of WWP1 and may mitigate the repressive effect of CDKN1B on MYC expression by degrading CDKN1B through ubiquitination.

CDKN1B expression was significantly reduced in PCa tissues (Fig. 6C), and CDKN1B expression was significantly negatively correlated with FAM84B, MYC, and WWP1 (Fig. 6D). PCa patients were divided into two cohorts: CDKN1B high expression (*n* = 20) and CDKN1B low expression (*n* = 18), based on the mean CDKN1B IHC scores in tumor tissues. Low expression of CDKN1B in tumor tissues was associated with higher Gleason score, T stage, and lymph node metastasis in patients (Table 6).

**Table 6:** Correlation between CDKN1B expression in tumor tissues and clinical parameters of PCa patients

Clinical characteristics		*n* = 38		CDKN1B IHC score		*p*-value
				High (*n* = 20)	Low (*n* = 18)	
Age (years)	≥ 66	17		8	9	0.5359
	< 66	21		12	9	
PSA (ng/mL)	≥ 9.67	13		7	6	0.9139
	< 9.67	25		13	12	
Gleason score	≤ 6	11		11	0	0.0007*
	3 + 4	10		7	3	
	4 + 3	9		2	7	
	≥ 8	8		0	8	
AR status	Positive	30		16	14	0.8668
	Negative	8		4	4	
p53 status	Positive	15		7	8	0.552
	Negative	23		13	10	
T stage	pT2	23		17	6	0.0042*
	pT3	13		3	10	
	pT4	2		0	2	
Lymph node metastasis	Absent		27	17	10	0.0457*
	Present		11	3	8	

Chi-square test was used to analyze the correlation between gene expression in PCa tumor tissues and clinical characteristics of patients. PCa: prostate cancer; PSA: prostate-specific antigen; AR: androgen receptor; IHC: immunohistochemistry; *p < 0.05

MYCi361 treatment promoted CDKN1B protein expression, while exogenous overexpression of WWP1 diminished CDKN1B protein expression (Fig. 6E). Neither MYCi361 treatment nor exogenous overexpression of WWP affected the mRNA expression of CDKN1B (Fig. 6F). Co-IP experiments observed an interaction between endogenous WWP1 and CDKN1B protein and observed an increase in the ubiquitination level of CDKN1B protein caused by overexpression of WWP1 (Fig. 6G). Overexpression of WWP1 led to a significant decline in the stability of CDKN1B protein and a higher rate of protein degradation (Fig. 6H).

### FAM84B promotes PCa by suppressing CDKN1B expression through MYC/WWP1 axis

**Fig. 7:**
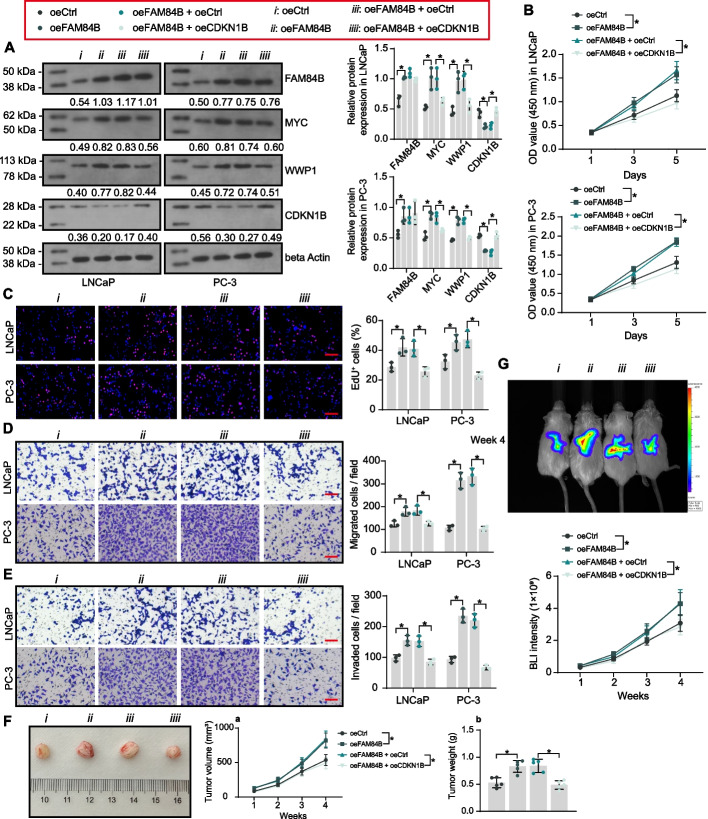
CDKN1B is an effector of FAM84B in PCa. PCa cells were treated with oeCtrl, oeFAM84B, oeFAM84B + oeCtrl, and oeFAM84B + oeCDKN1B. **A** FAM84B and CDKN1B protein expression in PCa cells was examined using western blot assays. **B**, **C** The PCa cell proliferation and DNA synthesis activity were assessed using CCK8 and EdU staining. **D**, **E** The migratory and invasive capacity of PCa cells were examined using transwell assays. **F** The growth rate of xenograft tumors formed by subcutaneously inoculated PC-3 cells in mice (*n* = 5). **G** Observation of tumor cell metastasis formed by intracardiac injection of PC-3 cells in mice by bioluminescence imaging (*n* = 5). Experiments were repeated three times with multiple wells. The bars indicate SD. *p < 0.05 (one-way/two-way ANOVA). Scale bar = 50 μm

CDKN1B was overexpressed in PCa cells stably overexpressing FAM84B. Overexpression of FAM84B induced the protein expression of MYC and WWP1 and repressed CDKN1B protein expression, as detected by western blot, whereas exogenous overexpression of CDKN1B significantly increased CDKN1B expression and repressed MYC and WWP1 expression (Fig. 7A). This result confirmed the negative feedback loop of MYC/WWP1/CDKN1B. Overexpression of FAM84B significantly activated the proliferation and DNA synthesis of PCa cells (Fig. 7B, C) and promoted cell migration and invasion (Fig. 7D, E), while CDKN1B expression blocked the oncogenic effect of FAM84B (Fig. 7B–E).

In in vivo experiments, PC-3 cells overexpressing FAM84B exhibited enhanced tumorigenic ability, with significantly increased tumor growth rate (Fig. 7F) and enhanced metastatic activity (Fig. 7G). In contrast, CDKN1B overexpression inhibited tumor development accelerated by FAM84B.

We also combined WWP1 knockdown intervention in PCa cells overexpressing FAM84B, and RT–qPCR detected that all three shRNAs effectively knocked down the mRNA expression of WWP1 in the cells without affecting CDKN1B mRNA expression (Fig. S4A). The shWWP1 1#, which had the best effect on WWP1 knockdown, was selected for subsequent assays. Western blot detected that the knockdown of WWP1 promoted protein expression of CDKN1B (Fig. S4B). The results of CCK8 and EdU assays showed that shWWP1-mediated knockdown of WWP1 significantly reduced the cell proliferation enhanced by FAM84B overexpression (Fig. S4C, D). Knockdown of WWP1 also inhibited PCa cell migration and invasion (Fig. S4E, F).

## Discussion

EccDNA is widespread in nearly half of human cancers, and the unique structure and molecular characteristics enable spatial and temporal plasticity of eccDNA functions, which regulates cancer initiation and progression [24]. For instance, a total of 200 eccDNA genes were obtained in breast cancer and among them, eccDNA-oriented ITGB7 was significantly upregulated in breast cancer patients and was associated with the menopause status of the patients [25]. However, the authors failed to dissect the detailed mechanism for eccDNA-oriented ITGB7. With this in mind, this study focused on FAM84B, which was found to be an eccDNA gene that is overexpressed in PCa. We hypothesized that it might play a similarly crucial role in PCa and could be a useful therapeutic target for PCa if its mechanism in this setting is better understood.

The overexpression of FAM84B was found to be correlated with a low survival rate in glioma patients [18]. Here, we identified the correlation between high FAM84B expression and higher Gleason score and advanced T stage in PCa. As for its functional role, the FAM84B gene was amplified and overexpressed in esophageal squamous cell carcinoma (ESCC) tissues, and the depletion of FAM84B reduced ESCC cell growth, migration, as well as invasion [26]. Consistently, Hsu et al. found that FAM84B protein was overexpressed in the majority of ESCC patients, and knockdown of FAM84B delayed tumor growth in ectopic xenografts [27]. Our assays yielded similar results, and we further presented that the oncogenic effects of FAM84B were related to the eccDNA since the knockdown of Lig3, the ligase that is responsible for the generation of eccDNA, contributed to the less malignant phenotype of PCa cells, which was overturned by FAM84B overexpression.

Using integrated bioinformatics prediction, we obtained WWP1 as the downstream target of FAM84B in PCa. WWP1, which was upregulated in PCa clinical specimens, was identified as a direct target of microRNA-452, and the knockdown of WWP1 inhibited the migration and invasion of PCa cells [28]. We also revealed the overexpression of WWP1 in PCa tissues and cells and its positive correlation with FAM84B expression, T stage, and Gleason score in PCa patients. How does FAM84B regulate the WWP1 expression in PCa then? Through analysis using the METABRIC and TCGA datasets, Homer-Bouthiette et al. provided evidence that MYC and FAM84B were frequently co-amplified in breast cancer [29]. Lee et al. also found that WWP1 was genetically amplified and frequently overexpressed in multiple cancers, including PCa, and WWP1 may be transcriptionally activated by the MYC proto-oncogene [30]. Sanarico et al. observed that WWP1 inactivation severely impaired the growth of primary acute myeloid leukemia blasts and cell lines in vitro and the leukemogenic potential of WWP1-depleted acute myeloid leukemia cells was reduced upon transplantation into immunocompromised mice [31]. Therefore, we wondered whether the regulatory effects of FAM84B on WWP1 in PCa were elicited through its partner MYC. Indeed, the inhibitor of MYC, MYCi361, reversed the enrichment of the WWP1 promoter pulled down using the antibody against MYC. MYCi361 has been found to suppress in vivo tumor growth in mice and augment tumor immune cell infiltration [14]. Here, we observed that the antitumor and antimetastatic effects of MYCi361 were mitigated by overexpression of WWP1, suggesting that WWP1 is a downstream target of MYC in PCa.

WWP1 is a member of the C2-WW-HECT E3 ligase family, and the association of some substrates can release autoinhibition-related domains and make WWP1 have polyubiquitination activity [32]. WWP1 has been reported to repress endogenous CDKN1B expression through ubiquitin–proteasome-mediated degradation since it had a strong preference for catalyzing the Lys-48-linked polyubiquitination of CDKN1B in vitro [33]. In the present study, we found CDKN1B as a substrate of WWP1 as well, and its protein expression was enhanced by MYCi361 and reduced by WWP1 overexpression. Faisal et al. identified that CDKN1B deletions were associated with metastasis in African American men with surgically treated PCa [34]. García-Gutiérrez et al. found that MYC inhibited the transcription of CDKN1B but also enhanced its degradation through the upregulation of components of ubiquitin ligase complexes at least in some cells [35]. Moreover, MYC has been summarized by Hydbring et al. to regulate the expression of several central cell cycle regulators, including CDK2, CDKN1B, and SKP2, which formed a triangular network to control each other, and these factors in turn modulate MYC through posttranslational modifications, including phosphorylation and ubiquitylation, impacting on its transcriptional activity on genes [36]. In the present study, we observed that the overexpression of FAM84B induced the expression of MYC and WWP1, thus repressing the expression of CDKN1B. CDKN1B overexpression, by contrast, reduced the expression of MYC and its target WWP1. Thus, we have confirmed the presence of a negative feedback loop of MYC/WWP1/CDKN1B. In addition, the oncogenic role of FAM84B in PCa cells was reversed by CDKN1B overexpression, indicating that CDKN1B was the effector of FAM84B in PCa. Considering WWP1 overexpression could not completely restore the full growth capacity of the cells in vitro and in vivo in the presence of MYC inhibition, more oncogenic pathways should be discovered in the future to dissect the effects of MYC in PCa.

## Conclusions

**Fig. 8:**
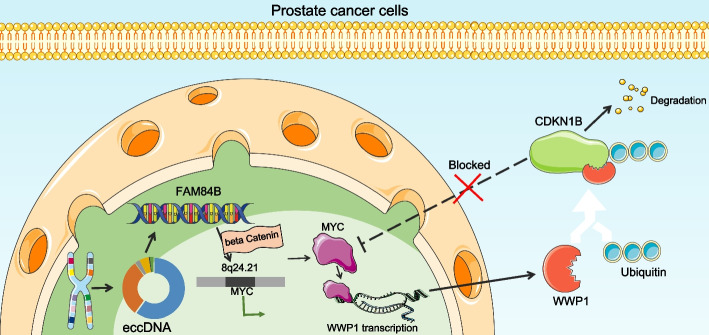
The mechanism of eccDNA-derived FAM84B regulation of PCa progression. The eccDNA in PCa promotes the expression of FAM84B through the intact transcript it carries. FAM84B promotes the transcriptional activation of MYC on WWP1 by inducing MYC that is coterminous with the 8q24.21 gene desert in a beta catenin-dependent manner and mediates the degradation of CDKN1B by WWP1. The repression of MYC expression by CDKN1B was alleviated by negative feedback regulation, thereby accelerating PCa progression

The present study showed the in vivo and in vitro effects of eccDNA-derived FAM84B on PCa. Moreover, FAM84B promoted cell growth and metastasis in PCa by negatively modulating the expression of CDKN1B through the MYC/WWP1 axis (Fig. 8). Thus, targeting eccDNA-derived FAM84B is promising as a novel therapeutic approach for PCa. Still, further large-sample investigations need to be conducted to determine the effects of any molecules in this axis on the prognosis of PCa patients and to verify our findings.

### Supplementary Information

Supplementary Material 1. Supplementary Fig. 1 The potential role of eccDNA in PCa and the transcripts carried. Supplementary Fig. 2 Co-localization of MYC with FAM84B at gene desert 8q24.21. Supplementary Fig. 3 FAM84B promotes MYC transcription in a beta Catenin-dependent manner. Supplementary Fig. 4 Knockdown of WWP1 inhibits FAM84B-enhanced malignant biological behavior of PCa cells.

Supplementary Material 2. Supplementary Table 1 eccDNA present in PCa. Supplementary Table 2 Complete gene transcripts of eccDNA 1#, 2# and 3#. Supplementary Table 3 Genes co-expressed with FAM84B in PCa.

## Data Availability

All data sets used or analyzed in this study are available from the corresponding author upon request.

## Abbreviations

eccDNA: Extrachromosomal circular DNA
PCa: Prostate cancer
WWP1: WW domain-containing protein 1
CDKN1B: Cyclin-dependent kinase inhibitor 1B
HPECs: Human prostate epithelial cells
FBS: Fetal bovine serum
