# Supplementary material for: Extrachromosomal circular DNA promotes prostate cancer progression through the FAM84B/CDKN1B/MYC/WWP1 axis
Source: Cell Mol Biol Lett. 2024 Jul 12;29:103. doi: 10.1186/s11658-024-00616-3 (PMC11245840; doi:10.1186/s11658-024-00616-3)
Supplement: Supplementary file 1 — Supplementary Material 1. Supplementary Fig. 1 The potential role of eccDNA in PCa and the transcripts carried. Supplementary Fig. 2 Co-localization of MYC with FAM84B at gene desert 8q24.21. Supplementary Fig. 3 FAM84B promotes MYC transcription in a beta Catenin-dependent manner. Supplementary Fig. 4 Knockdown of WWP1 inhibits FAM84B-enhanced malignant biological behavior of PCa cells. [file 11658_2024_616_MOESM1_ESM.docx]

**Supplementary Figure 1**

**
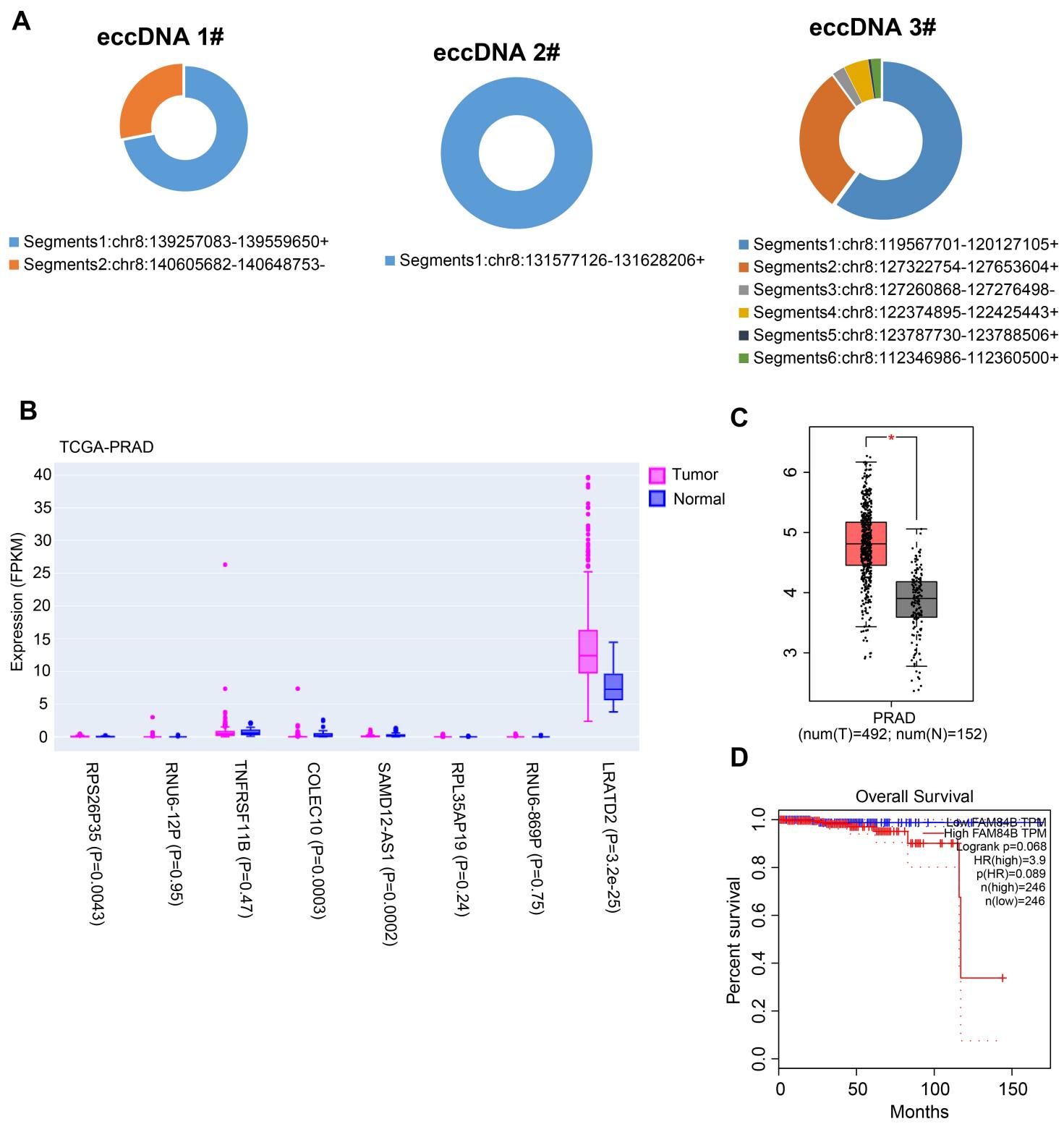
**

**Supplementary Figure 1** The potential role of eccDNA in PCa and the transcripts carried. (A) The presence of eccDNA in PCa. (B) Expression of the genes with complete transcript contained in eccDNA 3# in PCa. (C-D) The expression and the prognostic significance of FAM84B in RPAD were analyzed in GEPIA.

**Supplementary Figure 2**

**
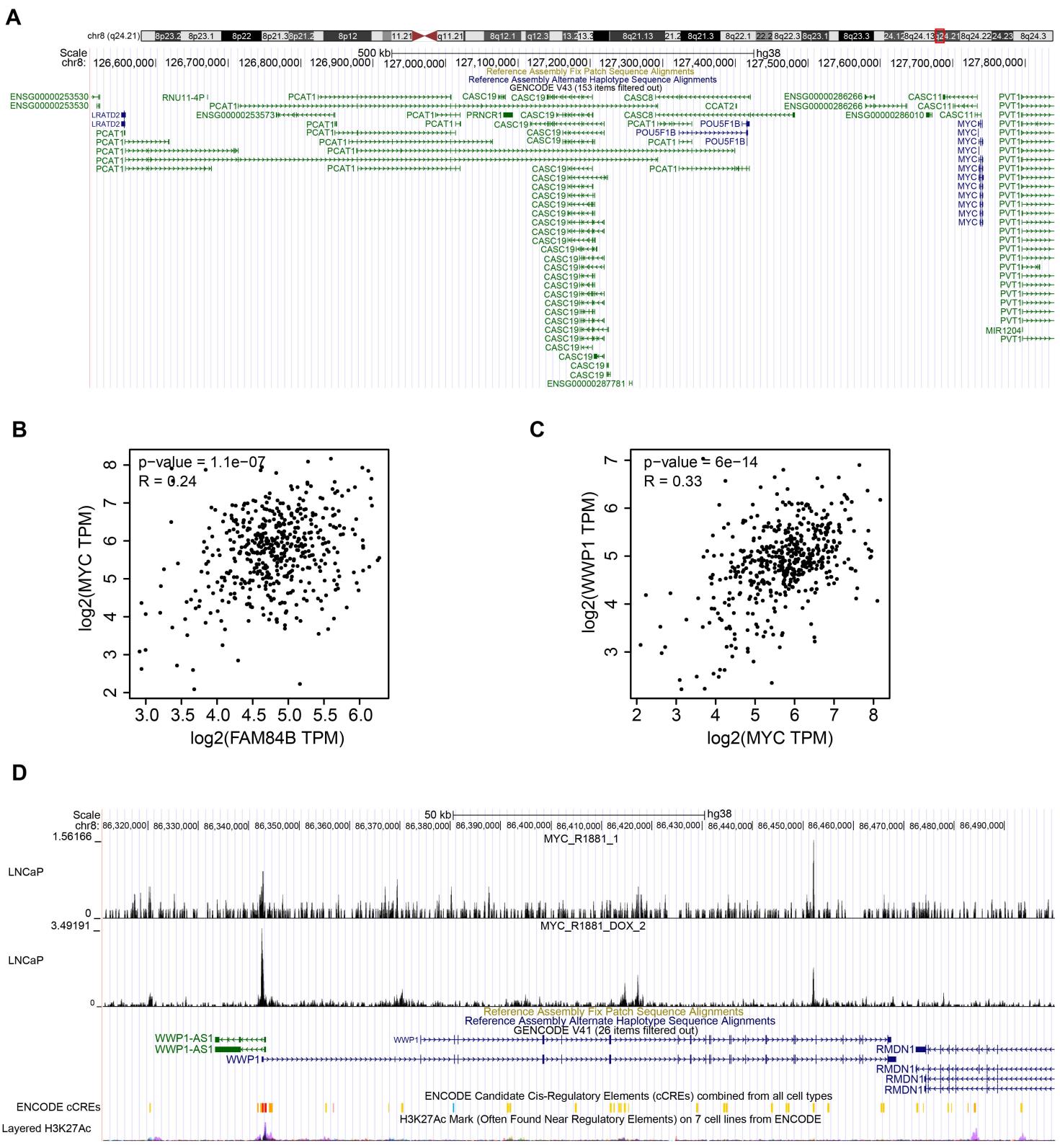
**

**Supplementary Figure 2** Co-localization of MYC with FAM84B at gene desert 8q24.21. (A) Genomic localization of FAM84B. (B) Correlation of MYC and FAM84B expression in PCa. (C) Correlation of MYC and WWP1 expression in PCa. (D) The ability of MYC to enrich the WWP1 promoter.

**Supplementary Figure 3**

**
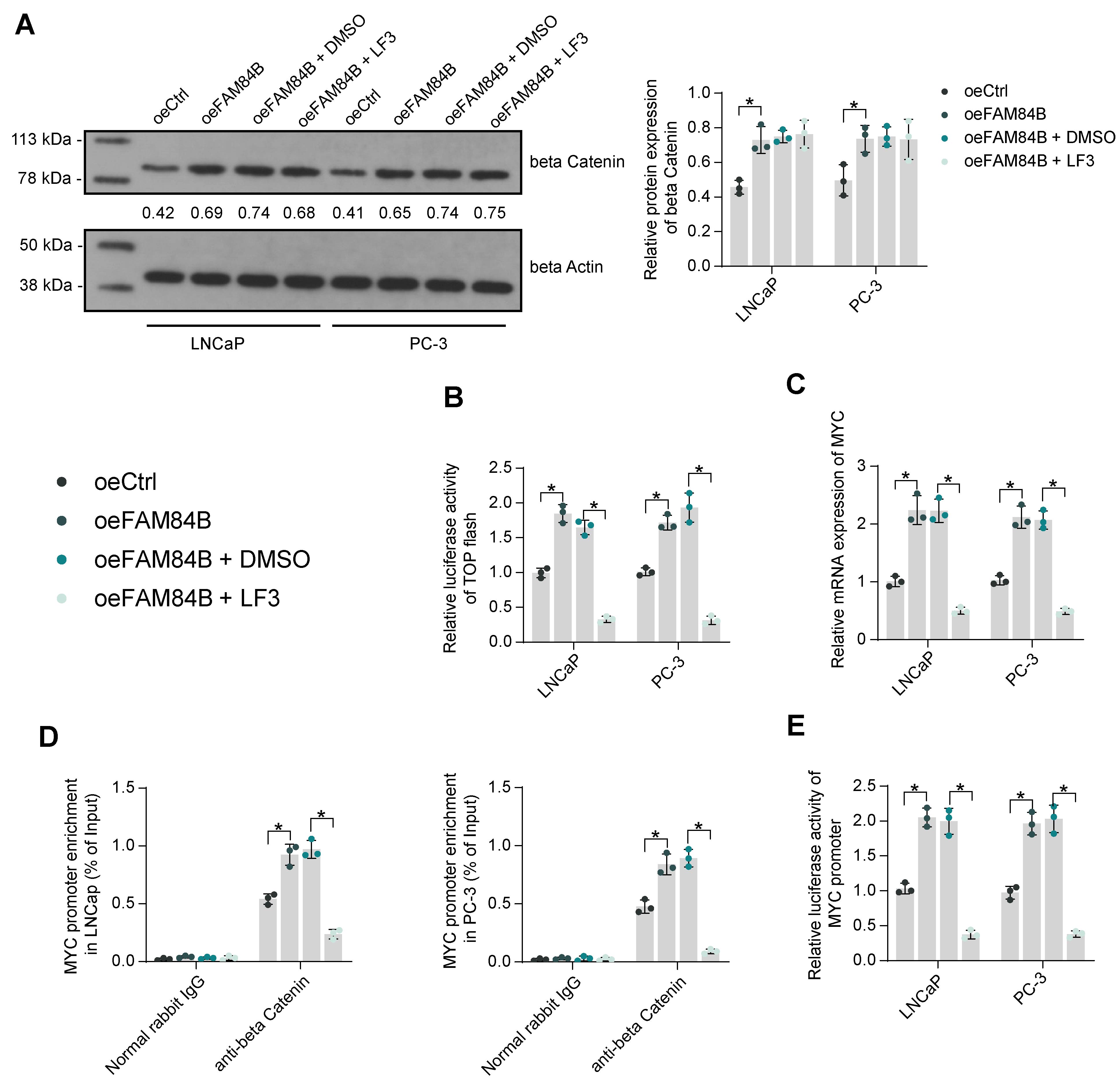
**

**Supplementary Figure 3** FAM84B promotes MYC transcription in a beta Catenin-dependent manner. (A) Effect of overexpression of FAM84B and LF3 treatment on beta Catenin protein expression in PCa cells was examined using western blot analysis. (B) Effect of overexpression of FAM84B and LF3 treatment on beta Catenin signaling was examined using TOP/FOP flash assay. (C) Effect of overexpression of FAM84B and LF3 treatment on MYC mRNA expression (transcript level) in PCa cells was examined using RT-qPCR. (D) Effect of overexpression of FAM84B and LF3 treatment on the binding of anti-beta Catenin at the MYC promoter was examined using ChIP-qPCR. (E) Effect of overexpression of FAM84B and LF3 treatment on the transcriptional activity of the MYC promoter was examined using dual-luciferase reporter assay. Experiments were repeated three times. The bars indicate SD. **p* < 0.05 (two-way ANOVA).

**Supplementary Figure 4**

**
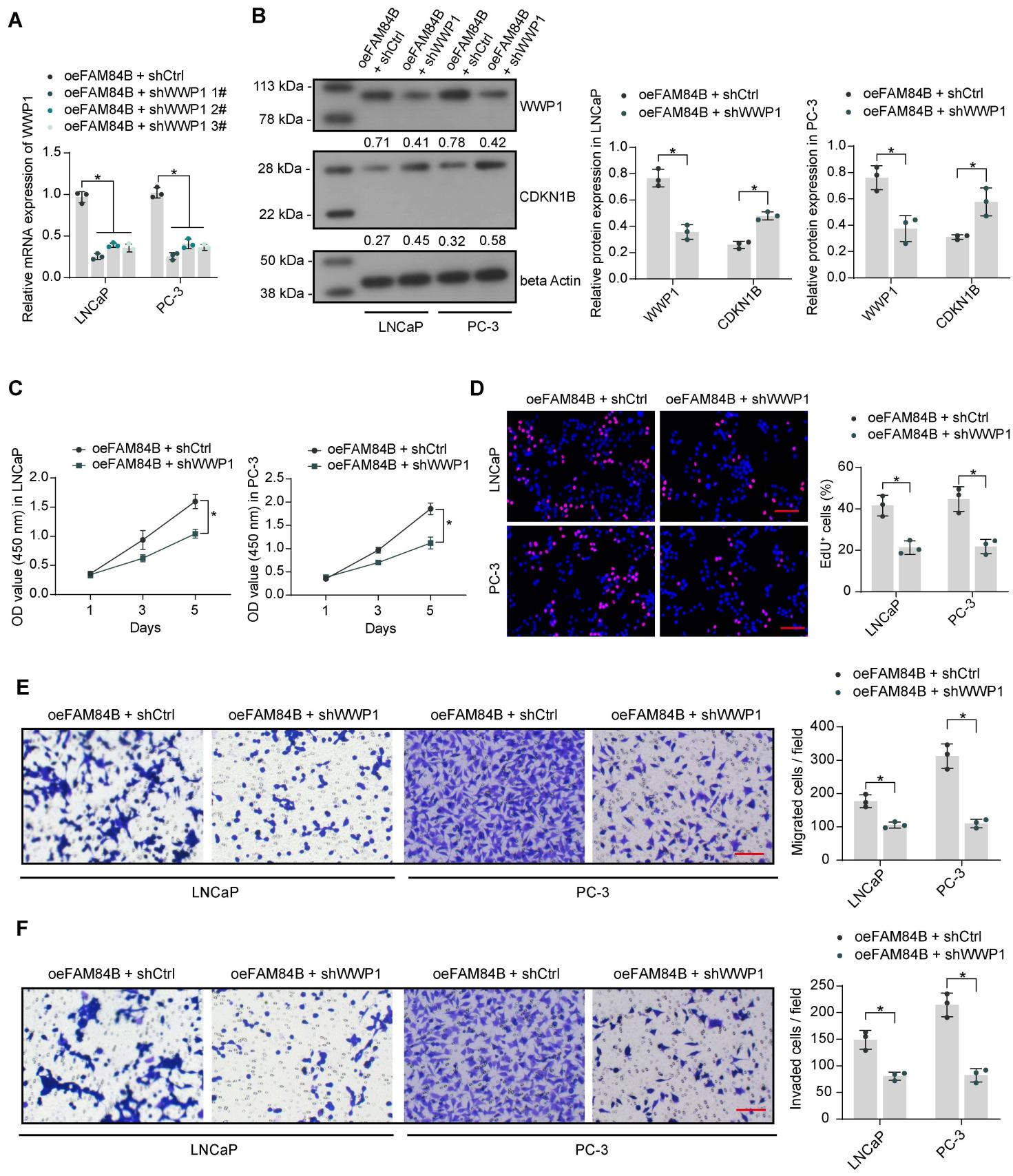
**

**Supplementary Figure 4** Knockdown of WWP1 inhibits FAM84B-enhanced malignant biological behavior of PCa cells. (A) shRNA knockdown efficiency for WWP1 was examined using RT-qPCR. (B) Effect of knockdown of WWP1 on CDKN1B protein expression in PCa cells was examined using western blot analysis. (C-D) The PCa cell proliferation and DNA synthesis activity were assessed using CCK8 and EdU staining. (E-F) The migratory and invasive capacity of PCa cells were examined using Transwell assays. Experiments were repeated three times. The bars indicate SD. **p* < 0.05 (two-way ANOVA).
