# Supplementary material for: Extrachromosomal circular DNA promotes prostate cancer progression through the FAM84B/CDKN1B/MYC/WWP1 axis
Source: Cell Mol Biol Lett. 2024 Jul 12;29:103. doi: 10.1186/s11658-024-00616-3 (PMC11245840; doi:10.1186/s11658-024-00616-3)
Supplement: Supplementary file 2 — Supplementary Material 2. Supplementary Table 1 eccDNA present in PCa. Supplementary Table 2 Complete gene transcripts of eccDNA 1#, 2# and 3#. Supplementary Table 3 Genes co-expressed with FAM84B in PCa. [file 11658_2024_616_MOESM2_ESM.docx]

**Supplementary Table 1** eccDNA present in PCa

| Number | ID | Segments | Unique Genes | Copy count | Sample type | Tissue | Disease | Cell line |
| --- | --- | --- | --- | --- | --- | --- | --- | --- |
|  |  |  |  |  |  |  |  |  |
| 80 | hsa_Chr8_2S_1 | chr8:139257083-139559650+ | 2 | 9.32 | Cancer cell line | Prostate | Prostate cancer (adenocarcinoma) | PC3 |
|  |  | chr8:140605682-140648753- |  |  |  |  |  |  |
| 81 | hsa_Chr8_1S_4 | chr8:131577126-131628206+ | 1 | 8.61 | Cancer cell line | Prostate | Prostate cancer (adenocarcinoma) | PC3 |
| 82 | hsa_Chr8_6S_1 | chr8:119567701-120127105+ | 18 | 6.66 | Cancer cell line | Prostate | Prostate cancer (adenocarcinoma) | PC3 |
|  |  | chr8:127322754-127653604+ |  |  |  |  |  |  |
|  |  | chr8:127260868-127276498- |  |  |  |  |  |  |
|  |  | chr8:122374895-122425443+ |  |  |  |  |  |  |
|  |  | chr8:123787730-123788506+ |  |  |  |  |  |  |
|  |  | chr8:112346986-112360500+ |  |  |  |  |  |  |
| 510 | hsa_Chr8_2S_10 | chr8:139257083-139559650+ | 2 | 9.08 | Cancer tissue | Prostate | Prostate cancer (adenocarcinoma) |  |
|  |  | chr8:140605682-140648753- |  |  |  |  |  |  |
| 511 | hsa_Chr8_1S_45 | chr8:131577126-131628206+ | 1 | 8.5 | Cancer tissue | Prostate | Prostate cancer (adenocarcinoma) |  |
| 796 | hsa_Chrx_1S_2 | chrX:72776425-72829524+ | 2 | -1 | TM | Prostate | Prostate cancer |  |
| 827 | hsa_Chr4_1S_4 | chr4:150177614-150399910+ | 3 | -1 | TP | Prostate | Prostate cancer |  |
| 868 | hsa_Chr8_1S_82 | chr8:96349075-96428315+ | 2 | -1 | TM | Prostate | Prostate cancer |  |
| 869 | hsa_Chr12_1S_6 | chr12:63610448-92256120+ | 420 | -1 | TM | Prostate | Prostate cancer |  |
| 870 | hsa_Chr18_1S_5 | chr18:56002692-56801139+ | 29 | -1 | TM | Prostate | Prostate cancer |  |
| 871 | hsa_Chr11_3S_3 | chr11:77645962-93795635+ | 767 | -1 | TM | Prostate | Prostate cancer |  |
|  |  | chr11:93800740-109287500+ |  |  |  |  |  |  |
|  |  | chr11:122845129-135006515+ |  |  |  |  |  |  |
| 872 | hsa_Chr1_1S_16 | chr1:58879932-59784978+ | 23 | -1 | TM | Prostate | Prostate cancer |  |
| 873 | hsa_Chr3_1S_10 | chr3:172402026-172429224+ | 3 | -1 | TM | Prostate | Prostate cancer |  |
| 878 | hsa_2Chr_2S_25 | chr8:58820648-95934225+ | 1671 | -1 | TP | Prostate | Prostate cancer |  |
|  |  | chr12:53810015-110855041+ |  |  |  |  |  |  |
| 1078 | hsa_Chr8_1S_93 | chr8:73461497-93119652+ | 281 | -1 | TP | Prostate | Prostate cancer |  |
| 1203 | hsa_3Chr_9S_1 | chr1:174471374-174471795+ | 1235 | -1 | TP | Prostate | Prostate cancer |  |
|  |  | chr6:131936629-132137050+ |  |  |  |  |  |  |
|  |  | chr7:13184448-33930585+ |  |  |  |  |  |  |
|  |  | chr7:35620457-42875308+ |  |  |  |  |  |  |
|  |  | chr7:47110039-47916514+ |  |  |  |  |  |  |
|  |  | chr7:85218431-89215935+ |  |  |  |  |  |  |
|  |  | chr7:90427861-105445614+ |  |  |  |  |  |  |
|  |  | chr7:131963996-132166648+ |  |  |  |  |  |  |
|  |  | chr7:134024053-146412507+ |  |  |  |  |  |  |

**Supplementary Table 2** Complete gene transcripts of eccDNA 1#, 2# and 3#

|  | eccDNA segment | Gene | Gene locus (strand) | Overlap size | eccDNA ratio | Gene ratio | Ensembl gene | Gene type | HGNC ID |
| --- | --- | --- | --- | --- | --- | --- | --- | --- | --- |
| eccDNA 1# |  |  |  |  |  |  |  |  |  |
|  | chr8:139257083-139559650 | FAM135B | chr8:139142266-139509504 (-) | 252421 | 0.83 | 0.69 | ENSG00000147724.12 | protein_coding | HGNC:28029 |
|  | chr8:139257083-139559650 | FAM135B | chr8:139142266-139509504 (-) | 252421 | 0.83 | 0.69 | ENSG00000147724.12 | protein_coding | HGNC:28029 |
|  | chr8:140605682-140648753 | KCNK9 | chr8:140613081-140716352 (-) | 35672 | 0.83 | 0.35 | ENSG00000169427.8 | protein_coding | HGNC:6283 |
|  | chr8:140605682-140648753 | KCNK9 | chr8:140613081-140716352 (-) | 35672 | 0.83 | 0.35 | ENSG00000169427.8 | protein_coding | HGNC:6283 |
| eccDNA 2# |  |  |  |  |  |  |  |  |  |
|  | chr8:131577126-131628206 | KB-1568E2.1 | chr8:131607545-131667958 (-) | 20661 | 0.4 | 0.34 | ENSG00000253656.1 | lncRNA | Unknown |
|  | chr8:131577126-131628206 | KB-1568E2.1 | chr8:131607545-131667958 (-) | 20661 | 0.4 | 0.34 | ENSG00000253656.1 | lncRNA | Unknown |
|  |  |  |  |  |  |  |  |  |  |
| eccDNA 3# |  |  |  |  |  |  |  |  |  |
|  | chr8:119567701-120127105 | RPS26P35 | chr8:119774095-119774440 (-) | 345 | 0.00062 | 1 | ENSG00000242206.2 | processed_pseudogene | HGNC:36229 |
|  | chr8:119567701-120127105 | RNU6-12P | chr8:119988752-119988858 (+) | 106 | 0.00019 | 1 | ENSG00000207334.1 | snRNA | Unknown |
|  | chr8:119567701-120127105 | RP11-278I4.1 | chr8:119994818-119995114 (-) | 296 | 0.00053 | 1 | ENSG00000254247.1 | processed_pseudogene | Unknown |
|  | chr8:119567701-120127105 | TNFRSF11B | chr8:119935796-119964124 (-) | 28328 | 0.051 | 1 | ENSG00000164761.9 | protein_coding | HGNC:11909 |
|  | chr8:119567701-120127105 | RP11-278I4.2 | chr8:120075181-120081021 (-) | 5840 | 0.01 | 1 | ENSG00000254278.1 | lncRNA | Unknown |
|  | chr8:119567701-120127105 | COLEC10 | chr8:120007691-120120694 (+) | 113003 | 0.2 | 1 | ENSG00000184374.3 | protein_coding | HGNC:2220 |
|  | chr8:119567701-120127105 | SAMD12-AS1 | chr8:119632737-119918394 (+) | 285657 | 0.51 | 1 | ENSG00000281641.3 | lncRNA | HGNC:30937 |
|  | chr8:119567701-120127105 | SAMD12 | chr8:119201694-119634351 (-) | 66650 | 0.12 | 0.15 | ENSG00000177570.15 | protein_coding | HGNC:31750 |
|  | chr8:122374895-122425443 | RPL35AP19 | chr8:122392377-122392705 (-) | 328 | 0.0065 | 1 | ENSG00000239872.1 | processed_pseudogene | HGNC:36631 |
|  | chr8:127260868-127276498 | LINC00861 | chr8:126871965-127305033 (-) | 15630 | 1 | 0.036 | ENSG00000245164.8 | lncRNA | HGNC:45133 |
|  | chr8:127322754-127653604 | RP11-65D17.1 | chr8:127337699-127341783 (+) | 4084 | 0.012 | 1 | ENSG00000244791.3 | lncRNA | Unknown |
|  | chr8:127322754-127653604 | RP11-103H7.2 | chr8:127509630-127512685 (+) | 3055 | 0.0092 | 1 | ENSG00000253220.2 | lncRNA | Unknown |
|  | chr8:127322754-127653604 | RNU6-869P | chr8:127517008-127517114 (+) | 106 | 0.00032 | 1 | ENSG00000207138.1 | snRNA | Unknown |
|  | chr8:127322754-127653604 | RP11-89K10.2 | chr8:127594876-127595251 (-) | 375 | 0.0011 | 1 | ENSG00000253543.1 | processed_pseudogene | Unknown |
|  | chr8:127322754-127653604 | RP11-103H7.1 | chr8:127486434-127504841 (+) | 18407 | 0.056 | 1 | ENSG00000253427.1 | lncRNA | Unknown |
|  | chr8:127322754-127653604 | LRATD2 | chr8:127564688-127570723 (-) | 6035 | 0.018 | 1 | ENSG00000168672.4 | protein_coding | HGNC:24166 |
|  | chr8:127322754-127653604 | RP11-103H7.3 | chr8:127518571-127534605 (+) | 16034 | 0.048 | 1 | ENSG00000253530.2 | lncRNA | Unknown |
|  | chr8:127322754-127653604 | PCAT1 | chr8:127568568-128431295 (+) | 85036 | 0.26 | 0.099 | ENSG00000253438.4 | lncRNA | HGNC:43022 |

**Supplementary Table 3** Genes co-expressed with FAM84B in PCa

| Genes | Pearson’s correlation coefficients |
| --- | --- |
| UTP23 | 0.81 |
| ZNF623 | 0.78 |
| KIAA1429 | 0.78 |
| ESRP1 | 0.78 |
| FAM91A1 | 0.77 |
| WWP1 | 0.77 |
| KIAA0196 | 0.77 |
| PHF20L1 | 0.77 |
| TAF2 | 0.77 |
| NBN | 0.76 |
| ZNF252 | 0.76 |
| C8orf83 | 0.76 |
| VCPIP1 | 0.76 |
| UBE2W | 0.75 |
| ARMC1 | 0.75 |
| ATP6V1C1 | 0.75 |
| DERL1 | 0.75 |
| CPNE3 | 0.75 |
| NUDCD1 | 0.75 |
| RAD21 | 0.75 |
| MTFR1 | 0.75 |
| RB1CC1 | 0.74 |
| AZIN1 | 0.74 |
| UBR5 | 0.74 |
| DPY19L4 | 0.74 |
| ZNF146 | 0.73 |
| ZHX1 | 0.73 |
| OTUD6B | 0.73 |
| TRMT12 | 0.72 |
| MAL2 | 0.72 |
| UBN1 | 0.72 |
| PEX2 | 0.72 |
| STAU2 | 0.72 |
| MTDH | 0.72 |
| SMARCC1 | 0.71 |
| DCAF13 | 0.71 |
| RBM12B | 0.71 |
| ATP13A3 | 0.71 |
| ATAD2 | 0.71 |
| C8orf37 | 0.7 |
| C11orf30 | 0.7 |
| ZFAND1 | 0.7 |
| UHRF1BP1 | 0.7 |
| RRM2B | 0.7 |
| UBXN2B | 0.7 |
| ARFGEF1 | 0.7 |
| LYPLA1 | 0.7 |
| ZBTB44 | 0.7 |
| ZNF420 | 0.7 |
| XPOT | 0.7 |
| YTHDF3 | 0.7 |
| PTK2 | 0.7 |
| NCOA2 | 0.7 |
